# Genetics in a Danish Common Variable Immunodeficiency Cohort

**DOI:** 10.1007/s10875-025-01896-w

**Published:** 2025-06-02

**Authors:** Camilla Heldbjerg Drabe, Mira Marie Laustsen, Hanne Vibeke Marquart, Hans Jakob Hartling, Rasmus L. Marvig, Jannik Helweg-Larsen, Ann-Brit Eg Hansen, Jens Lundgren, Marie Helleberg, Line Borgwardt, Terese L. Katzenstein

**Affiliations:** 1 Department of Infectious Diseases, Copenhagen University Hospital, Rigshospitalet, Esther Møllers Vej 6, Copenhagen Ø, 2100 Denmark; 2 Department of Genomic Medicine, Copenhagen University Hospital, Rigshospitalet, Copenhagen, Denmark; 3 Department of Immunology, Copenhagen University Hospital, Rigshospitalet, Copenhagen, Denmark; 4 Department of Clinical Medicine, Faculty of Health and Medical Sciences, University of Copenhagen, Copenhagen N, Denmark; 5 Department of Infectious Diseases, Copenhagen University Hospital, Hvidovre Hospital, Hvidovre, Denmark; 6 PERSIMUNE, Centre of Excellence for Personalised Medicine of Infectious Complications in Immune Deficiency, Copenhagen University Hospital, Rigshospitalet, Copenhagen, Denmark

**Keywords:** Common variable immunodeficiency, Inborn errors of immunity, Antibody deficiency, Whole exome sequencing, Whole genome sequencing, Next generation sequencing

## Abstract

**Purpose:**

Genetics of Common Variable Immunodeficiency (CVID) is complex and not fully elucidated. This study presents the clinical and genetic findings of a Danish CVID cohort and investigate whether initial genetic findings can be re-classified upon re-evaluation years later in time.

**Methods:**

From 2016 to 2021, individuals with CVID or a CVID-like-phenotype were examined using whole exome or whole genome sequencing in combination with comprehensive gene-panels. The results were re-evaluated to ensure up-to-date American College of Medical Genetics and Genomics (ACMG) classification after a median of 3.9 years. Further, a clinical-interpretation-algorithm is proposed.

**Results:**

Of 69 enrolled individuals, 57 met the current ESID-CVID-criteria of whom 29 (51%) had a genetic find. In total 67 ACMG class 3 to 5 variants were detected in 39 different genes. Class 3 variants (variants of uncertain significance (VUS)) accounted for 81% in the initial analysis. Upon re-evaluation 17 of 54 (31%) of the originally reported VUS were re-classified to a different ACMG-class or excluded. The developed clinical-interpretation-algorithm demonstrated high interobserver-agreement. A “definite/probable” disease causing (or contributing) genetic variant was found in 19% of the CVID-cohort and a “possible” in 18%.

**Conclusion:**

A genetic cause of CVID could be identified in a minority of CVID-individuals, whereas the majority had no or uncertain genetic findings. Re-evaluation of genetic results over time is recommended, though VUS remain a significant challenge in CVID-genetics. Therefore, continued research in both CVID-genetics and in non-genetic causes of CVID is needed.

**Supplementary Information:**

The online version contains supplementary material available at 10.1007/s10875-025-01896-w.

## Introduction

Genetics has advanced immensely in the last 10–15 years, due to increased availability of testing prompted by lower cost and better technologies, specifically Next-Generation Sequencing (NGS). As a result, an exponential increase in the discovery of monogenetic diseases has been achieved in the field of inborn errors of immunity (IEI) [1].

Common variable immunodeficiency (CVID) is the most common symptomatic IEI, with an estimated prevalence of 1:50.000–1: 25.000 [2–5]. CVID is characterized by hypogammaglobulinemia and variable clinical manifestations such as increased susceptibility to infections, autoimmunity, lymphoproliferation, and malignancies [6–10].

In recent years, several disease causing variants have been identified in CVID-individuals, especially in genes associated with B-cell activation and signaling, as well as immune-regulation [1, 11–14]. These gene-defects are not all causative of disease, some are considered disease predisposing, linked to disease severity, or to specific complications [11, 15, 16].

Genetic testing of CVID-individuals initially focused on specific groups, such as individuals with non-infectious co-morbidity or early onset [13, 17–19]. However, international studies of unselected cohorts are increasingly reported [12, 20–22]. A probable disease-causing variant have been identified in 20–50% of individuals with CVID [12, 20–23].

Monogenetic causes of CVID have been identified, however defects in numerous genes of many different cellular pathways can result in a CVID-phenotype. Hence, CVID is not perceived as one disease but as an umbrella diagnosis covering several disease entities. Currently CVID-genetics remains incompletely elucidated.

Knowledge of gene-function and pathogenicity of specific variants is increasingly accumulating, therefore re-evaluation of previously reported variants may enhance the diagnostic yield of prior genetic testing [24, 25].

In this three-phased-study we aimed to (1) elucidate the genetics of an unselected Danish CVID-cohort, (2) explore the value of re-evaluating previously reported variants, and (3) develop a clinical-interpretation-algorithm to facilitate a systematical translation of genetic results in a clinical setting.

In phase 1, we performed the initial genetic analysis of adult CVID-individuals. We used an explorative approach with whole exome or whole genome sequencing, combined with comprehensive gene panels focusing on genes associated with immunodeficiency.

In phase 2, we re-evaluated the reported variants from phase 1 after a median of 3.9 years, incorporating updated guidelines for variant classification, knowledge on genes and specific variants.

In phase 3, we developed a clinical-interpretation-algorithm to systematically evaluate the likelihood of the identified genetic variants being causal for the specific clinical phenotype. The algorithm is intended to be used in addition to the American College of Medical Genetics and Genomics (ACMG) classification [26]. It incorporates a genotype-phenotype-match and inheritance-zygosity-match, aimed to systematically assess the probability of the reported variant being cause of (or contributing to) the clinical manifestations of the individual.

## Methods

### Setting and Study Population

Individuals with known or suspected IEI were recruited for genetic testing from August 2016 to July 2021, at the Department of Infectious Diseases, Rigshospitalet, Copenhagen, Denmark. Genetic and immunological work-up was performed by Department of Genomic Medicine and Department of Immunology, Rigshospitalet.

The following inclusion criteria were used: Adult (≥ 18 years of age) individuals; with diagnosed or suspected CVID; monitored or referred for a second opinion; and able to provide written informed consent. Individuals were excluded if other causes of hypogammaglobulinemia could not be excluded. Clinical data were obtained by review of medical records and captured through REDCap [27, 28].

Results of genetic testing of 94 individuals with non-CVID IEI diagnosis have been described elsewhere [25].

### Diagnostic Criteria

The definitions of CVID has changed over time [4, 29]. All diagnosis were reviewed to ensure adherence with the current 2019 ESID working definitions of Primary Immunodeficiencies (PIDs) [4]. The diagnosis of CVID requires evidence of B-cell deficiency with either poor vaccine-responses or low levels of switched memory B-cells. If information of both elements were missing, patients were referred for a new lymphocyte subpopulation analysis. If the CVID diagnosis could not be confirmed, another appropriate IEI diagnosis was assigned upon multidisciplinary discussion, according to ESID criteria [4].

### Clinical Phenotype

Clinical characteristics included gender, family PID-history, infection history, and co-morbidities; bronchiectasis; splenomegaly; lymphoproliferation; autoimmunity/inflammatory complication; and/or malignant complications. Time of symptom onset was based on self-reported symptoms. The date of diagnosis was defined as the date when the immunological work-up concluded an IEI diagnosis. If this information was not available, an approximated date was estimated.

Four different phenotypes were used to characterize the patients, as defined by Chapel et al. [30]: Cytopenia; Polyclonal lymphoproliferation; Unexplained persistent enteropathy; and No other disease-related complications (infection-only). We modified the definition of unexplained persistent enteropathy as subjective gastrointestinal symptoms (mainly diarrhoea) for more than three months, with no identified infectious cause (as gastrointestinal biopsies were rarely obtained). The cohort were further grouped into two; infections-only and complex phenotype.

### Phase 1: Initial Genetic Analysis

In the period from August 2016– May 2018, study participants were investigated by whole exome sequencing (WES) (*n* = 29), thereafter by whole genome sequencing (WGS) (*n* = 23). From May 2020 to end of study in July 2021, the interpretation of whole genome data included copy number variation (CNV) analyses as standard (*n* = 15). In 11 cases, sequencing analyses were also performed on family members.

Variants were filtered by two locally developed gene panels. Panel PID-1 (457 genes) was used in the beginning of the study period (*n* = 58), replaced by panel PID-2 (665 genes) in October 2020 (*n* = 9). The two PID-panels include both known disease-associated genes, as well as candidate genes associated with signalling pathways or functions related to the immune system. In few cases additional genes were included i.e. if there were particular clinical manifestations or rare homozygote variants identified. The sequencing methods and both panels have previously been reported [25]. The panels can be found at the online repository (Table S1).

Variants were classified in accordance with ACMG [26]. Variants of class 3 (variants of uncertain significant (VUS)), class 4 (likely pathogenic), and class 5 (pathogenic) were reported.

Data-analysis and variant classification were conducted independently by two molecular biologist and/or clinical geneticists, followed by a discussion and consensus on interpretation.

### Phase 2: Re-evaluation of Genetic Variants

From January to December 2023, we conducted a re-evaluation of the previously reported variants. All variants were re-interpreted according to the current algorithm and guidelines for variant interpretation and any new knowledge of the variants and genes was discussed by a molecular biologist and a clinical geneticist. The following resources were used: Alamut^®^ Visual [31], ClinVar [32], GnomAD [33], OMIM [34], HGMD [35] and PubMed [36]. The original genetic data was not re-analysed. The re-evaluation phase was based solely on previously reported variants, in exception of one patient (C125) where we included the parents in a trio-analysis.

### Phase 3: Clinical Interpretation of Genetic Variants

The ACMG-classification of genetic variants is solely based on variant-specific pathogenicity. This classification is based on current available evidence and is independent of the clinical presentation of the tested individual. As per local guidelines class 3 to 5 variants are reported regardless of zygosity.

However, the clinical consequence of the variant is not fully encompassed by the ACMG-classification. In example in cases where individuals have a heterozygote pathogenic variant in a gene associated with recessive disease (carrier). In this case the variant is less likely to be the cause of disease in the individual. In other cases, variants may be identified in genes associated with a distinct phenotype not seen in the patient (e.g. *SH3BP2* and Cherubism). In these cases, the identified variant is also a less likely cause of disease.

We developed a clinical-interpretation-algorithm to systematically evaluate the clinical consequence of the reported variants for the specific individual. In other words; the probability of the reported variant being the cause of (or contributing to) the specific clinical manifestations of the individual.

The algorithm is based on three factors: ACMG classification; genotype-phenotype-match; and inheritance-zygosity-match. This allowed us to stratify the variants into three categories: definite/probable; possible; or less likely contributing to or cause of disease (Table 1).

The stratification was conducted independently by two reviewers (CHD and MML). In case of discordant scores, a third reviewer (LB) scored the variant.

**Table 1:** Clinical-interpretation-algorithm of reported variants

Clinical-interpretation-algorithm	Definite /Probable	Possible	Possible	Less likely
ACMG Class	3–5	3–5	3–5	3–5
Genotype-phenotype match	Yes	Yes	Maybe	No
Inheritance-zygosity match	Yes	Maybe/No	Yes/Maybe/No	Yes/Maybe/No

Inclusion of variant ACMG class, the genotype-phenotype-match and inheritance-zygosity-match allows for the classification of variants into three clinical interpretation categories: Definite/probable, possible or less-likely contributing/causing the patients clinical phenotype. ACMG: American College of Medical Genetics and Genomics

## Results

### Clinical Diagnosis

**Fig. 1:**
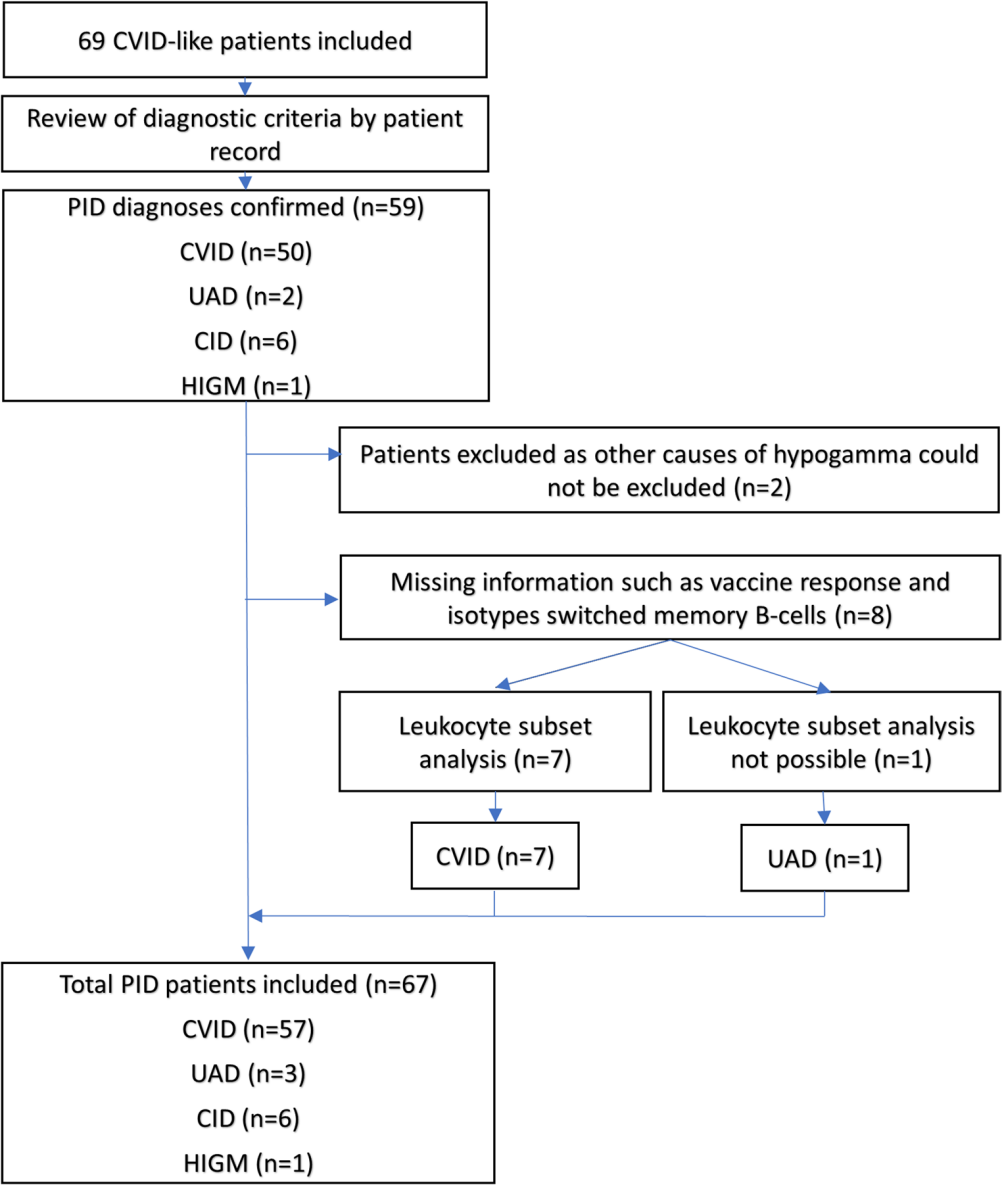
Patient flow. PID: Primary immunodeficiency, CVID: common variable immunodeficiency, UAD: unclassified antibody deficiency, CID: combined immunodeficiency, HIGM: Hyper-IgM-Syndrome

In total, 69 participants were included in the study. The participant flow is presented in Fig. 1. CVID-diagnosis was confirmed in 57 (83%) of the study participants. Two individuals were excluded because other causes of hypogammaglobulinemia could not be excluded. Three were classified as unclassified antibody deficiency (UAD) and six as having combined immunodeficiency (CID). One participant (C124) initially investigated for CVID/other IEI, was diagnosed with Hyper-IgM-Syndrome (HIGM) following the identification of a pathogenic variant in the CD40 ligand gene (described elsewhere [37]).

### Clinical Phenotype

Clinical characteristics of the cohort are presented in Table 2, stratified by diagnosis: CVID; UAD; and a combined category for the CID-patients and the HIGM-patient. Symptoms from childhood or early adulthood were common, but most patients were diagnosed in their second to fourth decade of life. The majority were of Danish origin, and only one individual (with UAD) reported parental consanguinity. The patients of non-Danish origin were from South-America (*n* = 2), Africa (*n* = 2), Asia (*n* = 1), or other European countries (*n* = 6).

**Table 2:** Cohort characteristics

	CVID	UAD	CID/HIGM
	*n* = 57	*n* = 3	*n* = 6/1
**Demographics**			
Female: n (%)	26 (46%)	0 (0%)	3 (43%)
Age: Median (IQR/*R)	43 (31, 59)	52 (44, 60)*	58 (38, 67)
Age at symptom onset: Median (IQR/*R)	21 (8, 31)	8 (4, 33)*	22 (19, 28)
Age at diagnosis: Median (IQR/*R)	33 (23, 43)	41 (27, 53)*	34 (29, 42)
Born in Denmark: n (%)	51 (89%)	2 (67%)	4 (57%)
Consanguinity: n (%)	0 (0%)	1 (33%)	0 (0%)
Family history of IEI: n (%)	6 (11%)	0 (0%)	0 (0%)
**Co-morbidity**			
Bronchiectasis: n (%)	22 (39%)	1 (33%)	4 (57%)
Splenomegaly: n (%)	18 (32%)	1 (33%)	5 (71%)
Lymphadenopathy: n (%)	9 (16%)	0 (0%)	4 (57%)
Autoimmunity: n (%)	29 (51%)	0 (0%)	5 (71%)
Malignancy: n (%)	8 (14%)	1 (33%)	3 (43%)
Hematological cancer: n (%)	2 (4%)	0 (0%)	2 (29%)
Solid cancer: n (%)	6 (11%)	1 (33%)	1 (14%)
**Clinical phenotype**			
Cytopenia: n (%)	6 (11%)	0 (0%)	0 (0%)
Polyclonal lymphoproliferation: n (%)	3 (5%)	0 (0%)	0 (0%)
Unexplained persistent enteropathy: n (%)	17 (30%)	0 (0%)	1 (14%)
No other disease-related complications: n (%)	25 (44%)	3 (100%)	2 (29%)
Two or more phenotypes: n (%)	6 (11%)	0 (0%)	4 (57%)
**Current therapy**			
IgRT total: n (%)	50 (88%)	3 (100%)	7 (100%)
Subcutaneous IgRT: n (%)	44 (77%)	2 (67%)	6 (86%)
Intravenous IgRT: n (%)	6 (11%)	1 (33%)	1 (14%)
Latest IgG: Median (IQR/*R)	8.0 (6.4, 9.6)	8.5 (8.0, 9.6)*	7.8 (6.1, 8.6)
Profylactic antibiotica: n (%)	12 (21%)	1 (33%)	1 (14%)
Current immune-modulation: n (%)	9 (16%)	0 (0%)	3 (43%)

CVID: common variable immunodeficiency, UAD: unclassified antibody deficiency, CID: combined immunodeficiency, HIGM: Hyper-IgM-Syndrome, IEI: Inborn errors of immunity, IQR: Interquartile range, R: range, IgRT: Immunoglobulin replacement therapy, IgG: Immunoglobulin G levels

Non-infectious complications were common, especially autoimmunity, which affected half of the CVID-group and five of six individuals with CID. Twelve individuals (18%) had malignancies.

When the CVID-cohort was categorised based on the modified criteria defined by Chapel et al. [30], 51 out of 57 (89%) individuals could be assigned to one specific phenotype (Table 2). The remaining six individuals had multiple complications.

### Phase 1: Genetic Results

We detected one or more ACMG class 3, class 4 or class 5 variants in 29 out of 57 (51%) of the CVID cohort and in nine of ten (90%) of the UAD/CID/HIGM-cohort (Table 3). A detailed description of each of these individuals’ phenotype can be found at the online repository (Table S2). Interestingly, 17 individuals (45%) had more than one variant identified.

**Table 3:** Summary table of genetic results of initial genetic analysis, after re-evaluation of genetic variants, and clinical interpretation of the variant

Genetic results	CVID	UAD	CID/HIGM
	*n* = 57	*n* = 3	*n* = 6/1
**Phase 1: Initial genetic analysis**			
Individuals with a class 3 to 5 variant: n (%)	29 (51%)	3 (100%)	6 (86%)
Total number of class 3 to 5 variants	55	4	8
VUS (Class 3): n (%)	45 (82%)	2 (50%)	7 (88%)
Likely Pathogenic (Class 4): n (%)	4 (7%)	0	0
Pathogenic (Class 5): n (%)	6 (11%)	2 (50%)	1 (12%)
Individuals with a definite molecular diagnosis: n (%)	2 (4%)	0	1 (14%)
**Phase 2: Re-evaluation of identified variants**			
Individuals with a class 3 to 5 variant: n (%)	27 (47%)	3 (100%)	6 (86%)
Total number of class 3 to 5 variants	45	4	7
VUS (Class 3): n (%)	31 (69%)	2 (50%)	4 (57%)
Likely Pathogenic (Class 4): n (%)	8 (18%)	0	2 (29%)
Pathogenic (Class 5): n (%)	6 (13%)	2 (50%)	1 (14%)
Individuals with a definite molecular diagnosis: n (%)	2 (4%)	0	1 (14%)
**Phase 3: Clinical interpretation of variants**			
Individuals with a			
Definite/probable genetic find: n (%)	11 (19%)	1 (33%)	4 (57%)
Possible genetic find: n (%)	10 (18%)	0 (0%)	1 (14%)
Less-likely genetic find: n (%)	6 (11%)	1 (33%)	1 (14%)

Definite molecular diagnosis is defined as identification of a pathogenic variant in a gene with established causality of disease and relevant zygosity. There was no change in definite molecular diagnoses after re-evaluation. CVID: common variable immunodeficiency, UAD: unclassified antibody deficiency, CID: combined immunodeficiency, HIGM: Hyper-IgM-Syndrome

**Fig. 2:**
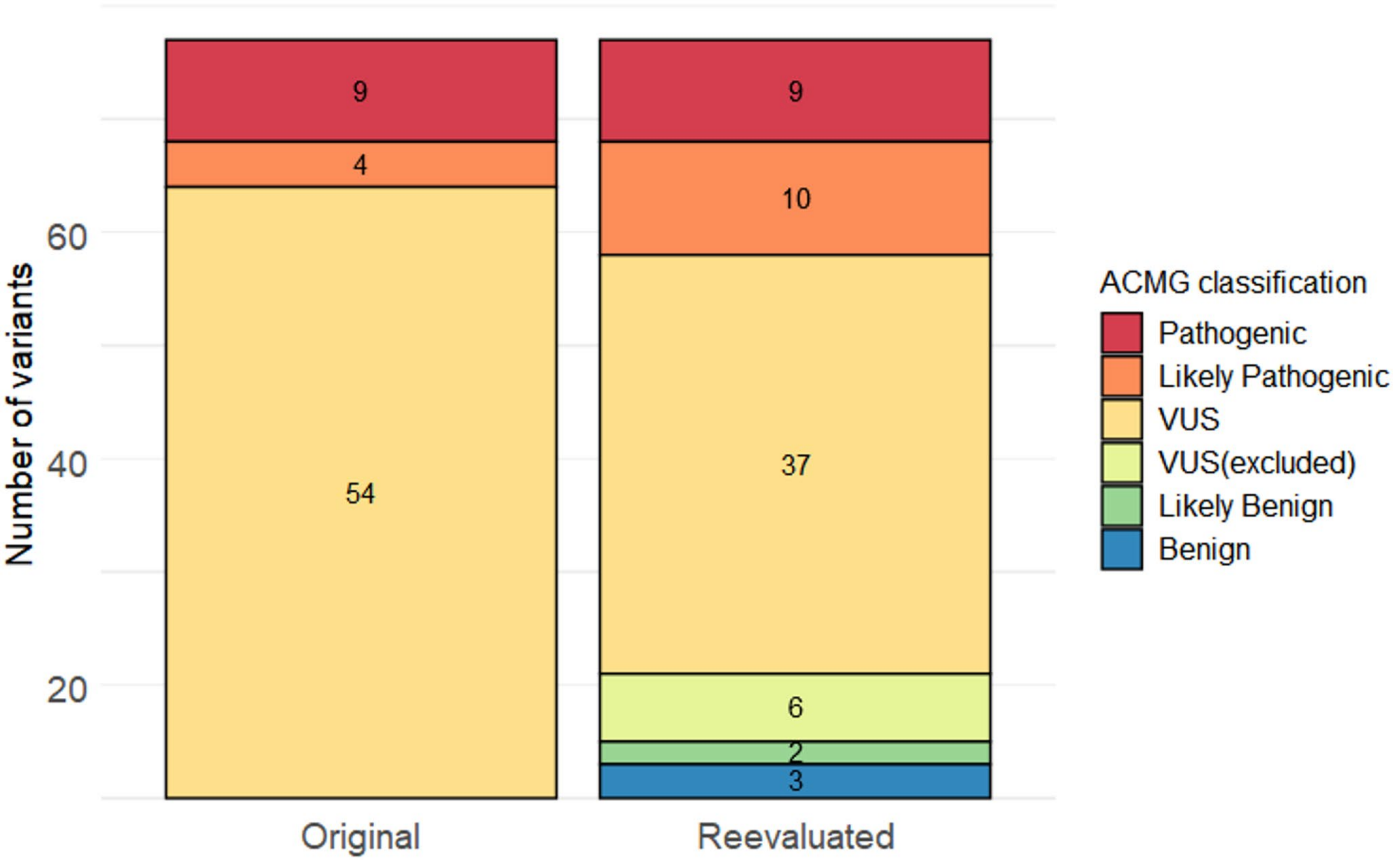
Number of variants identified in the entire cohort, stratified by ACMG-classification. The figure shows the ACMG-class-distribution of variants when originally reported, and after re-evaluation. Likely benign and benign variants were not originally reported. In total 17 variants of uncertain significance (VUS) were re-classified upon re-evaluation. These variants were classified as likely pathogenic (*n* = 6), likely benign (*n* = 2) or benign (*n* = 3). Six variants remained of uncertain significance but were excluded according to current reporting algorithm. ACMG: American College of Medical Genetics and Genomics

For the entire cohort a total of 67 variants were reported in 39 different genes. The variants are presented in Table 4. VUS constituted 81% of the identified variants (Table 3; Fig. 2). Of the 39 genes, 25 genes are listed in the International Union of Immunological Societies (IUIS) report of genes with known association to IEI [1].

**Table 4:** Specific genetic results of initial genetic analysis, after re-evaluation of genetic variants, and clinical interpretation of the variant

Gene	cNomen pNomen Transcript	Original ACMG class	Re-evaluated ACMG class	Clinical interpretation	Patient ID	Comment
**CVID**						
*TNFRSF13B* (n = 6)	c.542 C > A p.(Ala181Glu) NM_012452.2	Likely Pathogenic(*n* = 1) VUS (*n* = 2)	**Likely Pathogenic**	Definite/ Probable	C007, C058, C062	
	c.310T > C p.(Cys104Arg) NM_012452.2	Likely Pathogenic(*n* = 1) VUS (*n* = 2)	**Likely Pathogenic**	Definite/ Probable	C008, C025, C120	C008: The patient’s sister has the same variant, but do not share phenotype
*NFKB2* (n = 5)	c.2557 C > T p.(Arg853*) NM_001322934.2	Pathogenic (*n* = 2)	Pathogenic	Definite/ Probable	C003, C004	Patient C003 and C004 are related
	c.1093G > A p.(Gly365Arg) NM_001322934.2	VUS	VUS	Definite/ Probable	C024	It is not possible to determine if the two *NFKB2* variants is in cis/trans
	c.2167G > A p.(Asp723Asn) NM_001322934.2	VUS	VUS	Definite/ Probable	C024	
	c.1288 C > T p.(Pro430Ser) NM_001322934.2	VUS	VUS	Definite/ Probable	C125	Inherited from healthy mother
*UNC13D* (n = 4)	c.2346_2349del p.(Arg782Serfs*12) NM_199242.2	Likely Pathogenic	Likely Pathogenic	Less likely	C040	
	c.2542 A > C p.(Ile848Leu) NM_199242.2	VUS	VUS	Less likely	C010	It is not possible to determine if the two *UNC* variants is in cis/trans
	c.2983G > C p.(Ala995Pro) NM_199242.2	VUS	VUS	Less likely	C010	
	c.283_285dup p.(Glu95dup) NM_199242.2	VUS	**VUS (excluded)**	NA	C125	Inherited from healthy father
*SH3BP2* (n = 4)	c.1429 C > T p.(Arg477Trp) NM_001122681.2	VUS	**Benign**	NA	C024	
	c.1414 C > T p.(Arg472*) NM_001145856.1	VUS	VUS	Less likely	C036	
	c.776 C > T p.(Pro259Leu) NM_001145856.1	VUS	VUS	Less likely	C009	
	c.1534 C > T p.(Arg512Cys) NM_003023.4	VUS	**VUS (excluded)**	NA	C125	Inherited from healthy mother
*FANCA* (n = 3)	c.2029G > A p.(Val677Met) NM_000135.4	VUS	VUS	Less likely	C015	
	c.559G > A p.(Val187Ile) NM_000135.4	VUS	VUS	Less likely	C015	
	c.3570G > T p.(Gln1190His) NM_000135.4	VUS	VUS	Less likely	C049	
*NLRP2* (n = 2)	c.175 C > T p.(Leu59Phe) NM_001174081.2	VUS	VUS	Possible	C054	
	c.2758 C > G p.(Leu920Val) NM_001174081.2	VUS	VUS	Possible	C041	
*NLRC3* (n = 2)	c.1258 A > G p.(Met420Val) NM_178844.4	VUS	VUS	Possible	C052	
	c.2248 C > T p.(Arg750Trp) NM_178844.4	VUS	VUS	Possible	C054	
*IL17RA* (n = 2)	c.1174G > T p.(Val392Leu) NM_014339.6	VUS	VUS	Less likely	C055	Inherited from healthy mother
	c.371_380del p.(Arg124Profs*9) NM_014339.6	Pathogenic	Pathogenic	Less likely	C055	Inherited from healthy mother
*IL17F* (n = 2)	c.91 C > T p.(Arg31Trp) NM_052872.4	VUS	**VUS (excluded)**	NA	C125	Inherited from healthy father
	c.388G > A p.(Val130Ile) NM_052872.4	VUS	VUS	Less likely	C061	
*NLRP3*	c.2182 A > G p.(Ser728Gly) NM_004895.4	VUS	VUS	Possible	C002	The patient and family are reported in PMID: 35874679
*TLR10*	c.2314 C > T p.(Arg772*) NM_030956.3	VUS	VUS	Possible	C008	The patient’s sister has the same variant, but do not share phenotype
*SKIV2L*	c.2947G > A p.(Asp983Asn) NM_006929.4	VUS	VUS	Possible	C009	
*PDE1B*	c.1343 C > T p.(Ala448Val) NM_000924.3	VUS	**VUS (excluded)**	NA	C014 (HO)	
*CASP10*	c.1337 A > G p.(Tyr446Cys) NM_032977.3	VUS	**Benign**	NA	C014	Inherited from healthy father
*FAS*	c.-34 A > G NM_000043.5	VUS	**Benign**	NA	C014	Inherited from healthy father
*NBN*	c.657_661del p.(Lys219Asnfs*16) NM_002485.5	Pathogenic	Pathogenic	Less likely	C015	
*TCF3*	c.22G > T p.(Ala8Ser) NM_003200.5	VUS	VUS	Definite/ Probable	C022	
*TRAF3*	c.352 C > T p.(Arg118Trp) NM_145725.2	VUS	VUS	Possible	C040	
*RPSA*	c.563 C > T p.(Thr188Ile) NM_002295.5	VUS	VUS	Less likely	C040	
*MAGT1*	c.848 C > T p.(Thr283Met) NM_032121.5	VUS	**Likely Benign**	NA	C043 (HE)	
*TLR4*	c.842G > A p.(Cys281Tyr) NM_138554.4	VUS	VUS	Possible	C045	
*JAK3*	c.2630 C > T p.(Ala877Val) NM_000215.3	VUS	VUS	Less likely	C045	
*FANCM*	c.2714 A > T p.(Glu905Val) NM_020937.4	VUS	VUS	Less likely	C049	
*PRRC2A*	c.1430G > A p.(Arg477His) NM_004638.4	VUS	VUS	Possible	C050	
*TNFRSF13C*	c.475 C > T p.(His159Tyr) NM_052945.3	VUS	VUS	Possible	C051	
*PAX5*	c.280 + 1G > T NM_001280556.2	VUS	VUS	Possible	C113	
*IBTK*	c.1505G > A p.(Arg502Gln) NM_015525.4	VUS	VUS	Possible	C113	
*NFKBID*	c.454G > C p.(Glu152Gln) NM_139239.3	VUS	VUS	Possible	C113	
*IRF2BP2*	c.1330G > A p.(Ala444Thr) NM_182972.2	VUS	VUS	Definite/ Probable	C120	
*LRBA*	c.5519-1G > A NM_001199282.2	Likely Pathogenic	Likely Pathogenic	Possible	C125	Inherited from healthy mother
*ARHGEF4*	c.2330 C > T p.(Pro777Leu) NM_001367493.1	VUS	**VUS (excluded)**	NA	C125	Inherited from healthy mother
*PARN*	c.434 A > C p.(Gln145Pro) NM_002582.4	VUS	**VUS (excluded)**	NA	C125	Inherited from healthy father
*FLG * **(** **n = 2** **)**	c.1501 C > T p.(Arg501*) NM_002016.2	Pathogenic	Pathogenic	Definite/ Probable	C120	(for atopic dermatitis) It is not possible to determine if the two *FLG* variants is in cis/trans
	c.2282_2285del p.(Ser761Cysfs*36) NM_002016.2	Pathogenic	Pathogenic	Definite/ Probable	C120	
**UAD**						
*RFX5*	c.880 C > T p.(Arg294*) NM_001025603.2	Pathogenic	Pathogenic	Less likely	C001	
*IKZF1*	c.1047G > C p.(Gln349His) NM_006060.5	VUS	VUS	Definite/ Probable	C037	
*MEFV*	c.2084 A > G p.(Lys695Arg) NM_000243.2	VUS	VUS	Definite/ Probable	C037	(for Familial Mediterranean Fever)
*JAK2*	c.1849G > T p.(Val617Phe) NM_004972.4	Pathogenic	Pathogenic	NA - Somatic variant	C123	Known pathogenic variant associated with myeloproliferative disorders
**CID**						
*TNFRSF13B * **(n = 3)**	c.512T > G p.(Leu171Arg) NM_012452.2	VUS	VUS	Definite/ Probable	C019	
	c.542 C > A p.(Ala181Glu) NM_012452.2	VUS	**Likely Pathogenic**	Definite/ Probable	C047	
	c.310T > C p.(Cys104Arg) NM_012452.2	VUS	**Likely Pathogenic**	Definite/ Probable	C077	
*LRBA * **(n = 2)**	c.263 A > C p.(Glu88Ala) NM_006726.4	VUS	VUS	Possible	C005	
	c.217–21,029 A > T NM_006726.4	VUS	**Likely Benign**	NA	C005	
*IL17F*	c.254 C > T p.(Thr85Ile) NM_052872.4	VUS	VUS	Less likely	C006	The patient is previously reported in PMID: 32047491
*TLR2*	c.1667T > C p.(Ile556Thr) NM_001318787.1	VUS	VUS	Less likely	C006	
**HIGM**						
*CD40LG*	c.31 C > T p.(Arg11*) NM_000074.3	Pathogenic	Pathogenic	Definite/ Probable	C124 (HE)	(For HIGM) The patient is previously reported in PMID: 33013931

We identified most variants in *TNFRSF13B* and *NFKB2*. If re-evaluation of the reported variant led to altered ACMG classification, the new classification is highlighted in bold. The clinical interpretation of the variant is based on the parameters; ACMG classification, genotype-phenotype-match, and inheritance-zygosity-match (as specified in Table 1). Only ACMG class 3–5 are incorporated in this classification, why variants with a benign (class 1) og likely benign (class 2) classification are stated as NA. ACMG: American College of Medical Genetics and Genomics, VUS: variant of uncertain significance, CVID: common variable immunodeficiency, UAD: unclassified antibody deficiency, CID: combined immunodeficiency, HIGM: Hyper-IgM-Syndrome. HO: Homozygote, HE: Hemizogyte. If not otherwise specified, the variants are heterozygote

For the CVID cohort, 55 variants were reported in a total of 33 genes. Variants in *TNFRSF13B* (encoding TACI (the Transmembrane Activator and CAML Interactor)) were the most frequent observation (*n* = 6), followed by variants in *NFKB2* (nuclear factor kappa-B 2) (*n* = 5) (Table 4). Variants were predominantly identified in candidate genes (*n* = 15 (27%)) and genes related to Predominantly Antibody Deficiencies (PAD) (IUIS Table 3) (*n* = 14 (25%)).

In total, a definite molecular diagnosis (defined as a pathogenic variant in a gene with known association to disease, and with relevant zygosity) was identified in three cases: two related patients with NFKB2-deficiency (C003, and C004) and the patient with HIGM (C124).

### Phase 2: Variant Re-evaluation

The median time from the initial genetic analysis to variant re-evaluation was 3.9 years (range 1.6–6.8 years). Upon re-evaluation we were able to re-classify 17 variants (13 unique variants) (Fig. 2; Tables 3 and 4). All the re-classified variants were initially reported as VUS. The re-classifiable variants were found throughout the initial sequencing period, with a median and range identical to the overall variant re-evaluation time.

The re-classified variants were:

***TNFRSF13B***, **c.310T > C and c.542 C > A**: We identified three different variants in *TNFRSF13B*: c.310T > C (p.Cys104Arg), c.542 C > A (p.Ala181Glu), and c.512T > G (p.Leu171Arg). The c.310T > C and c.542 C > A variants are often found in PAD individuals [12, 13, 15, 18, 20, 38, 39]. With the current algorithm and guidelines these two variants were re-classified as likely pathogenic, as there are well-established in vitro and in vivo studies supporting damaging effects (PS3) [40–44] and in-silico analysis supporting pathogenic effect (PP3) [45]. Even though the allele frequencies for the variants are greater than expected for the disorder (BS1), the prevalence is significantly increased in affected individuals compared with the prevalence in controls (PS4) [15, 40].

***LRBA***, **c.217–21,029 A > T**: A deep intronic variant in the *LRBA* was initially reported. The variant has not previously been reported in the literature and is *without* predicted splice-effect (MaxEntScan, SpliceAI). With the current algorithm and guidelines, the variant would not have been reported and therefore was re-classified as likely benign.

***CASP10***, **c.1337 A > G; *FAS*, c.-34 A > G and *SH3BP2*, c.1429 C > T**: These variants were re-classified to benign due to the high allele frequencies in the population (4,2% in the Non-Finnish European population; 6,3% in the African population; and 1, 5% in the South American population, respectively in updated versions of gnomAD (gnomAD v2.1.1)).

***MAGT1***, **c.848 C > T**: The variant was functionally evaluated by use of NKG2D-expession on CD8-positive-T-cells and NK-cells [46], where the NKG2D-expression was found normal. Thus, we re-classified the variant from VUS to likely benign.

***PDE1B***,**c.1343 C > T**: *PDE1B* is not in our PID-gene panels. At the initial time of genetic testing of our PID patients (in 2016), we used an algorithm where rare homozygous variants identified was included regardless of whether the variant was in the PID panel. As such, this variant was initially reported. The function of *PDE1B* is less known. PDE1B1 are expressed on lymphocytes, and PDE1B2 on macrophages and lymphocytes. PDE1B1-expression have been found in lymphoblastoid and leukemic cell lines [47, 48]. We found no new information about this variant or the gene, therefore the variant remains a VUS. However, due to the limited knowledge of the gene the find is speculative, and we would not report it with the current algorithm and guidelines.

***IL17F***, **c.91 C > T;*PARN***, **c.434 A > C;*SH3BP2***, **c.1534 C > T;*UNC13D***, **c.283_285dup;** and ***ARGHEF4***, **c.2330 C > T**: A patient with CVID and Evans syndrome (C125), had a genetic analysis conducted by a corporate company (INVITAE) prior to inclusion for this study. Here, five variants were reported: One likely pathogenic (*LRBA*, c.5519-1G > A) and 4 VUSs (*IL17F*, c.91 C > T; *PARN*, c.434 A > C; *SH3BP2*, c.1534 C > T; and *UNC13D*, c.283_285dup). In addition, we identified two other variants of interest: one VUS in *NFKB2*, c.1288 C > T, which has previously been reported in a patient with DAVID syndrome [49], and one VUS in *ARGHEF4*, c.2330 C > T. The *ARGHEF4* is not in the PID-panel, but was included in the analysis as another missense variant in *ARGHEF4* had been reported in a patient with Evans syndrome [50]. Neither of the variants have been functionally evaluated. In the re-evaluation phase we chose to include the healthy parents in a trio-analysis. None of the variants were *de-novo*. We were, not able to re-classify any of the variants according to ACMG guidelines. However, the *IL17F*, c.91 C > T; *PARN*, c.434 A > C; *SH3BP2*, c.1534 C > T; *UNC13D*, c.283_285dup; and *ARGHEF4*, c.2330 C > T variants were interpreted as less likely relevant for the clinical phenotype and were inherited from a healthy parent, therefore they were excluded in the final genetic result.

Thus, re-evaluation of the variants reduced the proportion of VUS from 54 of 67 variants (81%) to 37 of 56 variants (66%). The total number of CVID-individuals with an identified ACMG class 3 to 5 variant was 27 of 57 individuals (47%), and 8 of 10 individuals (80%) in the UAD/CID/HIGM-group (Table 3). None of the reclassified variants led to altered diagnosis or treatment of the patient.

Class 3 to 5 variants were more often found in the CVID-participants with “infection-only” phenotype vs. “complex phenotype”, but this was not statistically significant (60% vs. 38%, *p* = 0.11 (Fishers exact test)). Neither was there any difference in the likelihood of identifying a class 3 to 5 variant between CVID-individuals with childhood onset (< 18 years) or adult onset ( > = 18 years) of symptoms (54% vs. 42%, *p* = 0.43), or when we compared this CVID/CVID-like cohort to the other IEI (or suspected IEI) cohort evaluated at our centre [25] (47% vs. 50%, *p* = 0.86).

### Phase 3: Clinical Interpretation of Genetic Results

Using the clinical-interpretation-algorithm (Table 1), concordance between the two independent reviewers was high (53 out of 56 variants, 95%). In the three discordant cases, a third reviewer scored the variants and determined the final clinical interpretation of the reported variants.

In total, 11 CVID-individuals (19%) had variants that scored “definite/probable” with the clinical-interpretation-algorithm, whereas another 10 CVID-individuals (18%) had variants that were “possible” cause or contributing factor to their CVID-phenotype. For the remaining 6 CVID-individuals (11%), where a class 3 to 5 variant had been identified, these findings were considered “less likely” disease contributing (Tables 3 and 4).

Other genetic findings included two pathogenic variants associated with atopic dermatitis (*FLG*, C0120), a VUS for familial Mediterranean fever (*MEFV*, C037) and one somatic pathogenic variant for myeloproliferative disorder (*JAK2*, C123). These findings were not considered to be the cause of or contributing to the immunodeficiency phenotype.

## Discussion

We investigated the genetics of a Danish CVID-cohort with whole exome or genome sequencing during a five-year-period, filtering variants with two extensive gene panels. The results were re-evaluated at a median of 3.9 years after initial analysis. We found at least one ACMG class 3 to 5 variant in approximately half of the cohort, with the majority being class 3 (VUS). Upon re-evaluation, 17 of 54 (31%) of the VUSs could be re-classified or excluded according to current algorithms and guidelines. Finally, we introduced a clinical-interpretation-algorithm, and thereby interpreted the genetic results as “definite/probable” cause or contributing factor for the specific clinical phenotype in nearly one fifth, and “possible” in another fifth of the CVID-cohort.

Our diagnostic yield with approximately 20% of our CVID-cohort having a “definite/probable” genetic cause, is similar to findings reported in PAD/CVID patients [12, 18, 21]: However, it is considerably lower than some other studies, reporting identifiable genetic variants in up to half of the patients [13, 20, 22, 23, 51]. Nevertheless, direct comparison between different genetic studies of CVID (and other IEIs) is difficult in the absence of gold standard methodology for testing and reporting. The literature encompasses individuals investigated with diverse methods, such as Sanger sequencing, targeted NGS-panels, WES or WGS. There is also great variation in the gene-panels used, ranging from a few selected genes to the extensive panels used in this study and others [12, 14, 18, 20]. Further, the variant-interpretation step includes several different methods. Many adhere to the ACMG-guidelines, but other approaches are also used [20, 52, 53]. Finally, the diagnostic yield is significantly influenced by the interpretation of the variants, with some studies considering only definite/probable variants, while others include possible variants as well.

Beyond the differences in methodology, differences in population composition also vary. In cohorts with greater proportion of consanguinity, the likelihood of identifying an underlying genetic cause of disease is increased [20] and substantial geographical variations are reported [13, 20].

As in other PAD cohorts, variants in *TNFRSF13B* were the most frequent finding in this study. *TNFRSF13B* encodes TACI; a receptor of B cell activating factor (BAFF) and A Proliferation Inducing Ligand (APRIL) mainly expressed on B- and plasma cells. TACI is an important regulator of immune cell homeostasis, differentiation, and activation [16]. TACI variants are found in 5–10% of PAD and CVID cohorts. As in our cohort, the Cys104Arg and Ala181Glu variants accounts for the vast majority. These variants are significantly increased in frequency in CVID-cohorts compared to controls [12, 15, 18, 20, 38, 39]. TACI variants were initially suspected causal for CVID [54, 55], but is now perceived as disease modifying e.g. increasing the risk of CVID or predisposing to autoimmune and lymphoproliferative complications [15, 16]. TACI variants are frequently found in the general population and in healthy family members [15, 55, 56], suggesting incomplete penetrance or the need for a second hit (genetic or environmental) to be pathogenic. TACI variants are difficult to classify according to the ACMG-classification, as variants with incomplete penetrance and risk-alleles are not easily classified in the scoring system. Thus, it is debated how to best classify these variants [11, 16]. The new classification recommendations from the ClinGen Low Penetrance/Risk Allele Working Group addresses exactly this challenge and propose a novel classification [57], where these TACI variants could be classified as “established risk alleles”.

The second most frequent finding in our study was variants in *NFKB2*, which is also a common find in other CVID/PAD-cohorts [23]. Contrary, we did not identify any variants in *NFKB1* in our cohort. *NFKB1* was early recognised as one of the most common genetic causes of CVID [58]. This could be a local genetic phenomenon, but more likely due to local referral mechanisms. In Denmark, PAD patients are cared for by infectious disease specialists. The clinical appearance of *NFKB1-*deficient patients is often dominated by inflammatory manifestations [59], thus *NFKB1-*deficient patients may not be recognised as PAD individuals and therefore not referred to our centre.

The importance of re-evaluation of the reported variants over time is demonstrated by our study, where multiple VUS could be reclassified, although this did not result in changes in diagnosis or treatment of patients. Genetic knowledge is continuously accumulating generated by the increasing use of genetics worldwide. This includes understanding of gene-function, specific variant characteristics, or simply better knowledge of allele frequencies in the general population [60]. However, re-evaluation is time-consuming and resource demanding. We were able to re-classify variants from the entire study period, including the most recent results 1,5 years prior to re-evaluation. Thus, an appropriate time-interval for re-evaluation of genetic results is not evident. The need for re-evaluation is multifactorial, depending on settings and resources; the specific gene of interest; and the clinical situation of the patient. The threshold for re-evaluation should be lower in cases where a possible re-classification could have implications for treatment-strategies or genetic counselling.

We used the ACMG-classification for genetic analysis. This classification strictly relies on variant specific pathogenicity. We therefore aimed to facilitate systematic translation of genetic reports into the clinical setting by incorporating clinical information using the clinical-interpretation-algorithm (Table 1). In general, we found the algorithm simple to use and inter-observer variability was low. A challenge is that the algorithm is not without personal interpretation. For example, in cases where heterozygote variants were identified in genes associated with autosomal recessive disorders, where monoallelic variants have previously been reported in CVID/CVID-like individuals (though not proven causative). In these cases, it is difficult to exclude that there is a geno-phenotype match, or inheritance-zygosity match, although the evidence for the opposite may be very limited. Further, the gene-related phenotype may be biased by the initial description for the gene-defect. The *TRAF3* Arg118Trp variant, for example, was initially associated with herpes simplex encephalitis [61], and TRAF3 deficiency is therefore linked to herpes infections in major reference resources (OMIM 614849) [1]. However, the variant has since been increasingly recognised in European cohorts, and does not seem to be a highly penetrant cause of herpes simplex encephalitis [62]. Other *TRAF3* variants have recently been described as causes of TRAF3-haploinsufficiency - a phenotype of recurrent bacterial infections, autoimmunity, systemic inflammation, B cell lymphoproliferation and hypergammaglobulinemia [62]. This stresses the importance of keeping an open mind to genotype-phenotype expansion. The participant carrying the Arg118Trp variant in our cohort (C040) does not resemble any of the phenotypes, and the clinical impact of the identified variant is uncertain.

The strengths of this study include the very well-defined group of study participants, that strictly fulfil the ESID diagnostic criteria and with complete information on clinical phenotypes. The comprehensible gene-panels allowed us to identify variants in both known PAD/CVID-genes as well as in genes related to other IEIs and/or candidate genes. This improves the genetic hit-rate and allows for exploration of yet unknown disease mechanisms. The challenge of this approach is detection of variants with uncertain clinical implication. Proving or disputing the pathogenicity of these variants is a considerable undertaking. This area of IEI genetics faces a substantial backlog that will require a sustained global effort to overcome.

When re-evaluating the variants, we only evaluated reported variants and did not re-analyse the genetic data. We have previously described the value of re-analysis of genetics in another IEI-cohort [25], where applying the larger gene-panel as well as including CNV-analysis did lead to a small increase in detected variants possibly associated to disease. Unfortunately, we did not have the required resources to re-analyse the genetic data in this study, which could potentially have aided in identifying novel genetic causes of disease.

Although we continue to gain knowledge of IEI and CVID-specific genetics, several factors complicate interpretation. This includes factors such as variable disease presentation; complex inheritance patterns (including variable expressivity); di- or poly-genetics; and epigenetics [22, 63–67].

In conclusion, our study supports the use of genetic testing of individuals with CVID and highlights the importance of re-visiting prior genetic results over time. We introduce a clinical-interpretation-algorithm, helpful in systematically interpreting the likelihood of the ACMG-classified reported variants causing or contributing to the specific clinical phenotype. Even though genetics have improved our understanding of CVID, CVID is in most cases not a monogenetic disorder. CVID-genetics remains complex reflected by the multiple immunological pathway-disturbances that can lead to a CVID-phenotype in combination with the large proportion of VUS. Together this calls for continued research of both genetic and non-genetic causes for CVID-disease, and of specific monogenetic disease trajectory and targeted treatment-strategies.

## Electronic Supplementary Material

Below is the link to the electronic supplementary material.

Supplementary Material 1

## Data Availability

Data generated in this study is either published in the article or supplementary data, or sensitive. For any inquiries, please contact corresponding author.
