# Supplementary material for: Genetics in a Danish Common Variable Immunodeficiency Cohort
Source: J Clin Immunol. 2025 Jun 2;45(1):102. doi: 10.1007/s10875-025-01896-w (PMC12129841; doi:10.1007/s10875-025-01896-w)
Supplement: Supplementary file 1 — Supplementary Material 1 [file 10875_2025_1896_MOESM1_ESM.pdf]

Table S1: List of 665 genes in PID-2.
Genes added in PID-2 are in bold.

ABI3	***BACH2***	*CARD14*	*CD81*	*CORO1A*	*DNMT3B*	***FBF1***	***GIMAP6***	*IFNL3*	*IL21R*	*JAGN1*	*MAP3K7*	*NBN*	***OTULIN***
ACD	*BANK1*	***CARD8***	*CD8A*	*CR2*	*DOCK2*	***FBRS***	***GINS1***	*IFNL4*	***IL22***	***JAK1***	***MAPK8***	*NCF1*	*PADI4*
ACKR1	***BCL10***	*CARD9*	***CDC42***	*CREBBP*	*DOCK8*	*FCER1G*	*GJC2*	***IGHE***	*IL23A*	*JAK2*	*MASP1*	*NCF2*	*PALB2*
ACP5	*BCL11B*	***CARMIL2***	*CDCA7*	*CSF1R*	*DPP10*	*FCGR1A*	***GRHL2***	***IGHG1***	***IL23R***	*JAK3*	*MASP2*	*NCF4*	*PARN*
ACTB	*BLK*	*CASP1*	***CDH17***	*CSF2RA*	***DPP4***	*FCGR2A*	***GSN***	***IGHG2***	*IL2RA*	***KDM6A***	*MAVS*	***NCKAP1L***	***PARP1***
ADA	*BLM*	*CASP10*	*CDKN2B*	*CSF2RB*	*DSG1*	*FCGR2B*	*GTF2H5*	*IGHM*	***IL2RB***	***KIR3DL1***	*MBL2*	*NCSTN*	***PAX1***
ADA2	*BLNK*	*CASP8*	*CEBPE*	*CSF3R*	***EFL1***	*FCGR3A*	***GUCY2C***	***IGKC***	*IL2RG*	***KMT2A***	*MC2R*	***NFAT5***	***PAX5***
ADAM17	*BLOC1S6*	***CAVIN1***	*CEBPG*	***CST3***	*ELANE*	*FCGR3B*	***HAVCR2***	***IGLC1***	***IL31RA***	*KMT2D*	***MCM10***	***NFE2L2***	*PBX1*
ADAR	***BPIFA1***	***CCBE1***	*CFD*	*CTC1*	*ELF4*	*FCGRT*	*HAX1*	*IGLL1*	*IL36RN*	*KRAS*	*MCM4*	*NFKB1*	*PCCA*
ADRB2	***BRCA1***	*CCL11*	*CFH*	*CTLA4*	***EPCAM***	***FCHO1***	*HELLS*	*IKBKB*	***IL4R***	***LACC1***	***MED13L***	*NFKB2*	*PCCB*
AGA	*BRCA2*	***CCL2***	*CFHR1*	***CTNNBL1***	*EPG5*	*FCN3*	***HLA-B***	*IKBKE*	***IL6***	*LAMTOR2*	*MEFV*	*NFKBIA*	*PDCD1*
AICDA	*BRIP1*	***CCL3***	*CFHR2*	*CTPS1*	*ERAP1*	***FERMT1***	*HLA-C*	*IKBKG*	***IL6R***	***LAT***	***MICA***	***NFKBID***	*PEPD*
AIM2	*BTK*	*CCL8*	*CFHR3*	*CTSC*	***ERBIN***	*FERMT3*	***HLA-DQB1***	*IKZF1*	***IL6ST***	*LCK*	*MLPH*	*NFKBIL1*	*PGM3*
AIRE	*C1QA*	*CCR5*	*CFHR4*	***CXCL12***	*ERCC2*	***FGA***	*HLA-DRB1*	*IKZF3*	***IL7***	***LDLR***	***MOGS***	*NHEJ1*	*PHF11*
AK2	*C1QB*	*CD19*	*CFHR5*	***CXCR1***	*ERCC3*	*FLG*	***HMOX1***	*IL10*	*IL7R*	*LIG1*	***MPI***	*NHP2*	*PIGA*
ALG13	*C1QC*	*CD209*	*CFI*	*CXCR4*	***ERCC4***	***FNIP1***	*HNMT*	*IL10RA*	***INO80***	*LIG4*	*MPO*	*NKX2-5*	*PIK3CD*
ALOX5	*C1R*	*CD244*	*CFP*	*CYBA*	***ERCC6L2***	***FOXM1***	*HPS1*	*IL10RB*	*INSR*	*LPIN2*	***MR1***	*NLRC3*	***PIK3CG***
ALPI	*C1S*	*CD247*	*CFTR*	*CYBB*	***EXTL3***	*FOXN1*	*HPS4*	*IL12A*	***IRAK1***	*LRBA*	*MRE11*	*NLRC4*	*PIK3R1*
ALPK1	*C2*	*CD27*	*CGAS*	***CYBC1***	*F12*	*FOXP3*	*HPS6*	*IL12B*	*IRAK4*	*LRRC8A*	*MRTFA*	*NLRP1*	*PLA2G7*
AP1S3	*C3*	*CD28*	*CHD7*	***DBR1***	*FADD*	*FPR1*	***HTR1A***	*IL12RB1*	*IRF2BP2*	*LTA*	*MS4A1*	*NLRP12*	*PLCG2*
AP3B1	*sd*	*CD3D*	*CHUK*	***DCLRE1B***	*FANCA*	***FPR2***	***HTRA2***	***IL12RB2***	*IRF3*	*LTBP3*	*MSH5*	*NLRP2*	***PLEKHM1***
AP3D1	*C4B*	*CD3E*	***CIB1***	*DCLRE1C*	*FANCB*	***FPR3***	***HYOU1***	*IL13*	***IRF4***	*LTBR*	***MSH6***	*NLRP3*	*PLG*
APOA1	*C4BPA*	*CD3G*	*CIITA*	*DDX11*	*FANCC*	***FUT2***	*IBTK*	***IL17A***	*IRF5*	*LYST*	***MSN***	*NLRP7*	*PMM2*
APOA2	*C4BPB*	*CD4*	*CLCN7*	*DDX41*	*FANCD2*	***FAAP24***	*ICOS*	*IL17F*	*IRF7*	***LYZ***	*MST1*	*NOD2*	*PMS2*
APOC2	*C5*	*CD40*	*CLEC4D*	*DDX58*	*FANCE*	*G6PC1*	***ICOSLG***	*IL17RA*	*IRF8*	***MAD2L2***	*MTHFD1*	*NOP10*	*PNP*
APOC3	*C6*	*CD40LG*	***CLEC4M***	***DEF6***	*FANCF*	*G6PC3*	*IFI16*	*IL17RB*	***IRF9***	*MAGT1*	***MTPAP***	*NOS2*	***POLA1***
APOL1	*C7*	*CD46*	*CLEC6A*	*DHFR*	*FANCG*	*G6PD*	*IFIH1*	***IL17RC***	*ISG15*	*MAL*	*MVK*	*NRAS*	***POLD1***
APP	*C8A*	***CD48***	*CLEC7A*	***DIPK2B***	*FANCI*	***GAD1***	*IFITM3*	*IL18*	*ITCH*	***MAL2***	***MX1***	*NSMCE3*	***POLD2***
ARHGEF1	*C8B*	*CD55*	***CLPB***	*DKC1*	*FANCL*	***GATA1***	***IFNAR1***	***IL18BP***	*ITGAM*	*MALT1*	*MYD88*	***OAS1***	*POLE*
ARPC1B	*C8G*	*CD59*	*CNBP*	***DNAJC21***	*FANCM*	*GATA2*	***IFNAR2***	*IL1RN*	*ITGB2*	*MAN2B1*	*MYO5A*	***ODC1***	***POLE2***
ATM	*C9*	***CD70***	***COL7A1***	*DNASE1*	*FAS*	***GATA3***	*IFNG*	*IL2*	*ITK*	***MAN2B2***	*MYO5B*	*ORAI1*	***POLR3A***
ATP6AP1	*CA2*	*CD79A*	*COLEC11*	*DNASE1L3*	*FASLG*	*GFI1*	*IFNGR1*	*IL20RA*	***ITPKB***	*MANBA*	***MYSM1***	***OSMR***	***POLR3C***
B2M	*CARD11*	*CD79B*	*COPA*	***DNASE2***	***FAT4***	***GIMAP5***	*IFNGR2*	*IL21*	***IVNS1ABP***	***MAP3K14***	***NBAS***	*OSTM1*	***POLR3E***

Table S1: List of 665 genes in PID-2.
Genes added in PID-2 are in bold.

POLR3F	*RECQL4*	***SEC61A1***	***SRP54***	*THBD*	*TRAF3IP2*	*ZBTB24*							
POMP	***REL***	***SELPLG***	***SRP72***	*TICAM1*	*TRAF6*	***ZC3HC1***							
POT1	***RELA***	*SEMA3E*	*STAT1*	*TINF2*	*TREX1*	***ZFP36***							
PRF1	***RELB***	*SERAC1*	*STAT2*	*TIRAP*	***TRIM22***	***ZNF34***							
PRKCD	***RELN***	*SERPING1*	*STAT3*	*TLR2*	***TRIM69***	***ZNF341***							
PRKDC	*RET*	***SGPL1***	*STAT4*	*TLR3*	*TRNT1*								
PRPS1	***RFWD3***	***SH2B3***	*STAT5A*	*TLR4*	***TSPAN14***								
PSEN1	*RFX5*	*SH2D1A*	*STAT5B*	*TLR5*	*TTC37*								
PSENEN	*RFXANK*	*SH3BP2*	***STAT6***	*TLR7*	*TTC7A*								
PSMA3	*RFXAP*	*SH3BP5*	*STIM1*	*TLR8*	***TTR***								
PSMB10	*RHOH*	***SH3KBP1***	*STING1*	*TLR9*	***TUBGCP3***								
PSMB4	*RIPK1*	*SKIV2L*	*STK4*	*TMC6*	*TYK2*								
PSMB8	*RIPK3*	***SLC13A4***	***STN1***	*TMC8*	***UBA1***								
PSMB9	*RMRP*	*SLC22A4*	*STX11*	*TNF*	***UBE2T***								
PSMG2	*RNASEH2A*	*SLC29A3*	*STXBP2*	***TNFAIP3***	*UNC119*								
PSTPIP1	*RNASEH2B*	*SLC35A1*	*TANK*	*TNFRSF11A*	*UNC13D*								
PTCRA	*RNASEH2C*	*SLC35C1*	*TAP1*	*TNFRSF13B*	*UNC93B1*								
PTEN	*RNF168*	*SLC37A4*	*TAP2*	*TNFRSF13C*	*UNG*								
PTPN2	*RNF31*	*SLC39A4*	*TAPBP*	*TNFRSF1A*	*UPB1*								
PTPN22	***RNU4ATAC***	***SLC39A7***	*TAZ*	*TNFRSF4*	*USB1*								
PTPRC	***RORC***	*SLC46A1*	*TBK1*	***TNFRSF9***	***USP18***								
PYCARD	*RPSA*	***SLC7A7***	*TBX1*	***TNFSF11***	*VAV1*								
RAB27A	*RSPH9*	***SLP76(LCP2)***	***TBX21***	*TNFSF12*	*VPS13B*								
RAC2	*RTEL1*	*SLX4*	*TCF3*	***TNFSF13***	*VPS45*								
RAD51	***SAMD3***	*SMARCAL1*	*TCIRG1*	*TNFSF13B*	*WAS*								
RAD51C	***SAMD9***	***SMARCD2***	*TCN2*	***TNIP1***	*WDR1*								
RAG1	***SAMD9L***	***SNORA31***	*TERC*	***TOM1***	*WDR5*								
RAG2	*SAMHD1*	***SNX10***	*TERT*	***TOP2B***	*WIPF1*								
RANBP2	***SART3***	***SOCS1***	***TET2***	***TP53***	*WRAP53*								
RASGRP1	*SBDS*	*SOCS4*	*TFRC*	***TPP1***	*XIAP*								
RASGRP2	*SCGB1A1*	*SP110*	***TGFB1***	*TPP2*	***XRCC2***								
RBCK1	*SCGB3A2*	*SPINK5*	*TGFBR1*	*TRAC*	*XRCC6*								
RC3H1	*SCIMP*	***SPPL2A***	***TGFBR2***	*TRAF3*	*ZAP70*								

Table S2: Clinical data for patients with identified genetic variants.
F: female, M: male, CVID: common variable immunodeficiency, UAD: unclassified antibody deficiency, CID: combined immunodeficiency, HIGM: Hyper-IgM-Syndrome.

Record ID	Sex, age	Origin (if not Danish)	Symptom onset	Infections	Autoimmunity, malignancies, and other manifestations
**CVID**					
**C002**	M, 10-19		0-9	Recurrent sinopulmory infections, aseptisk meningitis	
**C003**	F, 20-29	South-America	0-9	Recurrent sinopulmory infections, EBV (symptomatic infection)	Alopecia
**C004**	M, 40-49	South-America	0-9	Recurrent sinopulmory infections	Psoreasis
**C007**	M, 20-29		0-9	Recurrent sinopulmory infections, Otitis media, Invasive fungal infection (Aspergillus)	Thrombocytopenia, Hemolytic anemia, Neutropenia Splenomegaly, Hepatomegaly, Reduced lung function, Bronchiectasis
**C008**	M, 30-39		10-19	Recurrent sinopulmory infections, Otitis media	Splenomegaly, Bronchiectasis, Gastro-intestinal symptoms
**C009**	F, 50-59		20-29	No infections	Inflammatory bowel disease
**C010**	F, 40-49		10-19	Recurrent sinopulmory infections, EBV (symptomatic infection)	Thrombocytopenia, Hemolytic anemia, Hodgkin's lymphoma, Lymphadenopathy, Splenomegaly, Factor 5 Leiden Heterozygote
**C014**	F, 30-39		0-9	Recurrent sinopulmory infections	Thrombocytopenia, Neutropenia Lymphadenopathy, Splenectomy, Granulomatous disease, Liver fibrosis
**C015**	M, 60-69	Europe	20-29	Recurrent sinopulmory infections, CMV (symptomatic infection), Recurrent C. difficile	Pernicious anemia, Inflammatory bowel disease, Autoimmune thyroid disease, ATCH insufficiency Bronchiectasis, Gastro-intestinal symptoms, Osteoporosis
**C022**	M, 50-59		50-59	Recurrent sinopulmory infections	Thrombocytopenia, Splenomegaly
**C024**	M, 30-39		0-9	Recurrent sinopulmory infections, Otitis media	Thrombocytopenia, Reduced lung function, Bronchiectasis
**C025**	F, 70-79		40-49	Recurrent sinopulmory infections	Reduced lung function, alfa-1-antitrypsin deficiency
**C036**	F, 40-49		30-39	Recurrent sinopulmory infections	Autoimmune thyroid disease , Basal cell carcinoma

Table S2: Clinical data for patients with identified genetic variants.
F: female, M: male, CVID: common variable immunodeficiency, UAD: unclassified antibody deficiency,
CID: combined immunodeficiency, HIGM: Hyper-IgM-Syndrome.

Record ID	Sex, age	Origin (if not Danish)	Symptom onset	Infections	Autoimmunity, malignancies, and other manifestations
**C040**	M, 20-29		0-9	Recurrent sinopulmory infections	Splenomegaly
**C041**	F, 60-69		0-9	Recurrent sinopulmory infections, Otitis media	Inflammatory bowel disease
**C043**	M, 40-49		0-9	Recurrent sinopulmory infections	Inflammatory bowel disease, Splenomegaly, Bronchiectasis, Lactose intolerance
**C045**	M, 50-59		0-9	Recurrent sinopulmory infections	Diabetes Mellitus (type I), Primary biliary cirrhosis, Splenomegaly, Bronchiectasis
**C049**	M, 50-59		10-19	Recurrent sinopulmory infections	Gastrointestinal cancer, Melanoma, Reduced lung function, Bronchiectasis
**C050**	M, 30-39	Europe	20-29	Recurrent sinopulmory infections, Otitis media	Thrombocytopenia, Splenomegaly
**C051**	F, 60-69		40-49	Recurrent sinopulmory infections, Herpes labialis	Pernicious anemia, Autoimmune thyroid disease, Psoreasis Bronchiectasis, Nephrotic syndrome
**C052**	M, 30-39	Asia	10-19	Recurrent sinopulmory infections	Splenomegaly, Bronchiectasis, Gastro-intestinal symptoms, Hyperhidrosis
**C054**	F, 40-49		40-49	Recurrent sinopulmory infections, Organ abscess, HIV, HCV (cured)	Gastro-intestinal symptoms
**C055**	F, 20-29		20-29	Giardia	Gastro-intestinal symptoms
**C058**	F, 60-69		50-59	Recurrent sinopulmory infections	Gastro-intestinal symptoms
**C061**	F, 20-29		20-29	Prolonged fever	
**C062**	F, 10-19		10-19	Recurrent sinopulmory infections	
**C113**	M, 20-29		0-9	Recurrent sinopulmory infections, Otitis media	B-cell Lymphoma, Lymphadenopathy, Splenomegaly, Bronchiectasis
**C120**	M, 30-39		10-19	Recurrent sinopulmory infections	Atopic dermatitis
**C125**	M, 30-39		20-29	Recurrent sinopulmory infections	Thrombocytopenia, Hemolytic anemia Lymphadenopathy, GLILD, Splenomegaly, Bronchiectasis, Gastro-intestinal symptoms
**UAD**					

Table S2: Clinical data for patients with identified genetic variants.
F: female, M: male, CVID: common variable immunodeficiency, UAD: unclassified antibody deficiency,
CID: combined immunodeficiency, HIGM: Hyper-IgM-Syndrome.

Record ID	Sex, age	Origin (if not Danish)	Symptom onset	Infections	Autoimmunity, malignancies, and other manifestations
**C001**	M, 50-59		0-9	Recurrent sinopulmory infections, Viral encephalitis, Norovirus	Splenomegaly, Bronchiectasis, Gastro-intestinal symptoms
**C037**	M, 30-39	Africa	0-9	Recurrent sinopulmory infections, M. tuberculosis infection	
**C123**	M, 60-69		50-59	Recurrent sinopulmory infections	Basal cell carcinoma , Reduced lung function
**CID**					
**C005**	F, 70-79		10-19	Recurrent sinopulmory infections	Thrombocytopenia, Neutropenia Lymphadenopathy, Splenomegaly, Splenectomy, Reduced lung function, Bronchiectasis, Encephalitis (brain-biopsy with perivascular lymphocyte infiltration)
**C006**	F, 30-39	Europe	20-29	Recurrent sinopulmory infections, Otitis media	Juvenile rheumatoid arthritis, Gastrointestinal cancer, Lymphadenopathy, Splenomegaly, Hepatomegaly, Gastro-intestinal symptoms, Esophageal varices
**C019**	F, 40-49	Africa	30-39	Recurrent sinopulmory infections, Helicobactor pylori, Warts	Thrombocytopenia, Inflammatory bowel disease, Lymphadenopathy, Splenomegaly, Bronchiectasis, Gastro-intestinal symptoms
**C047**	M, 60-69		30-39	Recurrent sinopulmory infections	Small malignant B-cell clone, probably Mantle cells (Never treated, diagnosed two decades after the CVID diagnosis), Splenectomy, Bronchiectasis Depression
**C077**	M, 70-79		10-19	Recurrent sinopulmory infections	Inflammatory bowel disease, Autoimmune thyroid disease
**HIGM**					
**C124**	M, 20-29	Asia	10-19	Mucocutaneous Leishmaniasis	Splenomegaly
